# Hemidystonia secondary to pediatric thalamic glioblastoma: a case report

**DOI:** 10.1007/s00381-022-05698-y

**Published:** 2022-10-12

**Authors:** Filipe Wolff Fernandes, Assel Saryyeva, Philipp Ertl, Joachim Kurt Krauss

**Affiliations:** Department of Neurosurgery, Hannover Medical School, Carl-Neuberg Strasse 1, 30625 Hannover, Germany

**Keywords:** Glioblastoma, Hemidystonia, Hemiparesis, Stereotactic biopsy, Thalamus

## Abstract

**Introduction:**

Thalamic tumors are rare and uncommonly manifest as movement disorders, including hemidystonia. Despite this association, little is known about the evolution of hemidystonia.

**Case description:**

We report on a 11-year-old boy who complained of hypaesthesia and fine motor problems in the left hand. A magnetic resonance imaging showed a large mass in the right thalamus. Stereotactic biopsy revealed a WHO grade 4 astrocytoma, and the patient underwent normofractioned radiochemotherapy with proton-beam radiation and temozolomide. Three months later, a spastic hemiparesis developed on the left side, which progressed over months. Over the following months, the hemiparesis slowly improved, but hemidystonia in the same side developed. This was accompanied with radiological evidence of tumor regression, showing a persistent lesion in the ventral posterolateral and the intralaminar thalamus.

**Conclusion:**

This case illustrates the unusual and complex temporal course of appearance and disappearance of hemidystonia along with the regression and growth in glioblastoma involving the thalamus.

**Supplementary Information:**

The online version contains supplementary material available at 10.1007/s00381-022-05698-y.

## Introduction

Thalamic tumors account for about 1% of all intracranial tumors, appearing only rarely as glioblastoma [1]. Movement disorders are quite uncommon in such tumors, occurring in only 1–9% of patients [2–4]. Hemidystonia typically is the manifestation of a structural lesion in the basal ganglia or in the thalamus developing only months or years after the lesion [3, 5–8]. Although cerebral tumors have been identified as a possible cause for hemidystonia, little is known about the evolution of this rare manifestation of dystonia [3, 9, 10].

Here, we report on the transient appearance of hemidystonia in a child with a thalamic glioblastoma and its complex relationship both with tumor growth and hemiparesis.

## Case report

**Fig. 1:**
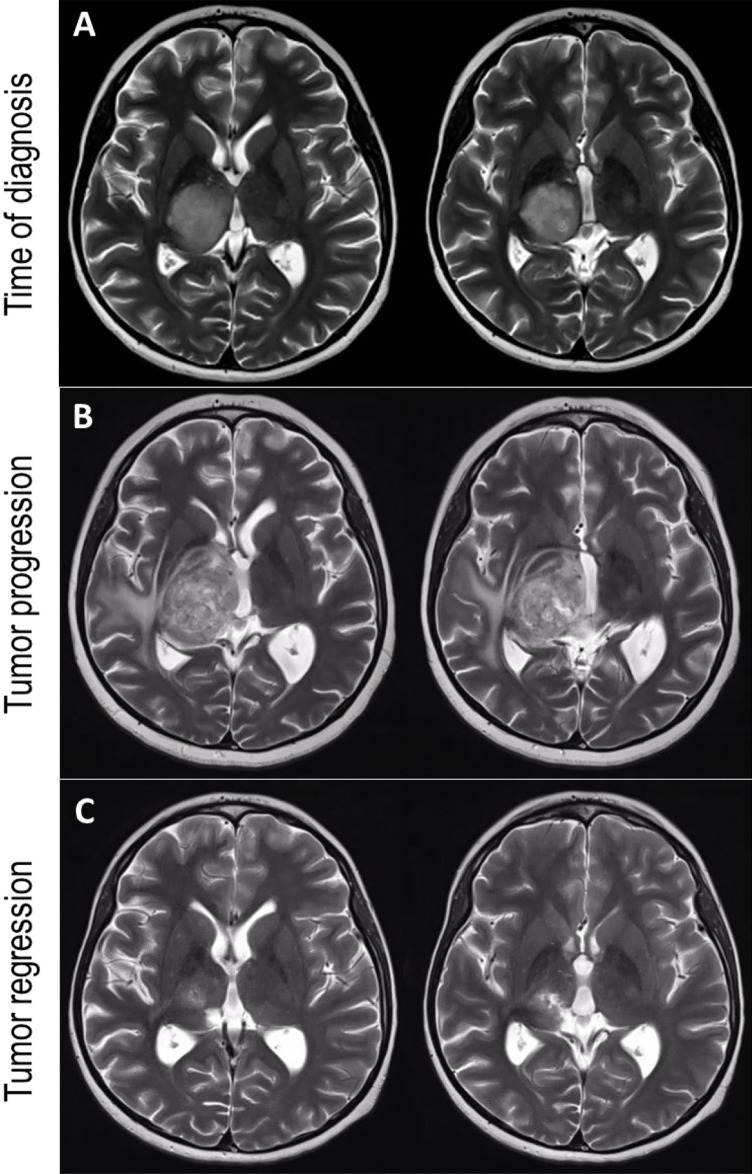
Development of a thalamic glioblastoma in an 11-year-old boy. T2-weight axial MRI scans show the tumor at different stages. **A** The tumor appears as a hyperintense lesion in the right thalamus with a local mass effect. **B** At 6 months post-stereotactic biopsy, there is a marked enlargement of the tumor with involvement of the posterior limb of the internal capsule, and accompanying edema paralleled by a progressive left-sided spastic hemiparesis. **C** At 16 months post-biopsy, there is a marked regression of the tumor with a lesion affecting the ventral posterolateral and intralaminar thalamus paralleled by improvement of the hemiparesis and development of left-sided hemidystonia

A 11-year-old right-handed boy complained of headaches and nausea, followed by hypaesthesia in the left hand and fine motor problems. Magnetic resonance imaging (MRI) showed a large mass (28 × 30 × 35 mm) in the right thalamus (Fig. 1A). The tumor was enhancing inhomogeneously after application of gadolinium. Stereotactic biopsy revealed a WHO grade 4 astrocytoma, IDH1/2 wild-type, MGMT unmethylated. Concomitant radiochemotherapy with proton beam radiation (54.9 Gy) and temozolomide was administered.

Three months later, a spastic hemiparesis developed on the left side, which was progressive over months, and the patient subsequently needed assistance with a wheel chair. He continued to take temozolomide according to monthly cycles. A MRI 6 months post-biopsy revealed tumor enlargement (39 × 30 × 43 mm) with tumor extension to the subthalamic region and compression of the internal capsule with massive edema (Fig. 1B).

Over the following months, the hemiparesis slowly improved, but hemidystonia developed. A MRI 16 months post-biopsy showed tumor regression (18 × 19 × 14 mm) with a focal lesion in the ventral posterolateral and the intralaminar thalamus (Fig. 1C). The patient continued treatment with temozolomide. The hemidystonia was more evident when walking or performing daily activities as shown in the video (Online Resource 1). At 26 months post-biopsy, the Burke-Fahn-Marsden dystonia rating scale motor score was 28, and the disability score was 9. Family history for dystonia was negative. Over the next 2 months, the tumor recurred along with an increase of the left-sided hemiparesis and concurrent subsidence of hemidystonia.

## Discussion

It has always been puzzling why tumors of the basal ganglia and thalamus present rarely with dystonia, tremor, or chorea, while discrete lesions in particular certain areas of the structures pertaining to the cortico-basal ganglia-thalamic-cortical circuitry are well known to underlie the development of movement disorders [5, 6]. In our case, the unusual temporal course of appearance and disappearance of hemidystonia along with the changes in imaging nevertheless allows to draw several conclusions.

While movement disorders may rarely be caused primarily by the tumor affecting the subcortical sensorimotor loop [2–4], they may also appear subsequently to specific treatment measures including surgery, chemotherapy, or targeted radiotherapy [3, 4]. This has been exemplified by a recent study of Sadighi et al. reporting two patients in whom dystonia became manifest only after removal of thalamic tumors in a cohort of 83 patients [4]. Of note, in our case dystonia became manifest only after radiotherapy lead to a marked reduction of the tumor volume but resulting in a thalamic lesion.

Previous studies have shown that hemidystonia most often is associated with lesions of the anterior putamen often combined with lesions of the caudate, while thalamic lesions have been observed more rarely [5]. The most common manifestation of dystonia secondary to thalamic lesions is focal hand dystonia is occasionally associated with sensory deficits, but few cases of hemidystonia have been described as well [6, 7]. While various sites in the thalamus have been described to be involved, our case clearly demonstrates a discrete lesion affecting both the ventral posterolateral and the intralaminar thalamus.

In several instances, as of hemidystonia described earlier, a pre-existent or concomitant hemiparesis was an accompanying feature indicating involvement of the posterior limb of the internal capsule [7]. While it is possible that the phenomenological appearance of dystonia is simply overshadowed by the presence of motor weakness, it has also been postulated that dystonia may be the result of faulty reorganization of subcortical circuitries in the presence of lesions in the sensorimotor loop. The dynamics in our case suggests that both are relevant.

The prognosis of movement disorders associated with pediatric thalamic tumors is quite variable [3, 4]. The effect of medication is limited [3, 4]. The primary concern is to obtain control of tumor growth. As shown here, the later may be the primary determinant for the dynamics of the movement disorders in such cases.

## Supplementary Information

Below is the link to the electronic supplementary material.

Supplementary file1 Online Resource 1—Hemidystonia after the improvement of hemiparesis of the left side. First segment of the video corresponds to presentation 20 months post-biopsy, and the second segment at 26 months post-biopsy. (MP4 2822 KB)

## Data Availability

Upon reasonable request.
